# Study on the microcrystal cellulose and the derived 2D graphene and relative carbon materials

**DOI:** 10.1038/s41598-023-48393-x

**Published:** 2023-12-27

**Authors:** Si-Yu Long, Jin-Lei Liu, Ling-Qiang Zhou, Wen-Da Lv, Xue-Quan Xian, Pei-Duo Tang, Qi-Shi Du

**Affiliations:** 1 National Key Laboratory of Non-food Biomass Technology, National Engineering Research Center for Non-food Biorefinery, Guangxi Academy of Sciences, Nanning, 530007 Guangxi China; 2 Fujian Yuanfu Biomass Technology Co., Ltd., Jiangle, Sanming, 353300 Fujian China

**Keywords:** Chemical engineering, Environmental chemistry, Materials chemistry, Chemistry, Materials science

## Abstract

Microcrystal cellulose (MCC) is a green and sustainable resource that widely exists in various lignocellulose species in percentage 10% to 30%. The fine powder of MCC is often discarded in industrial productions that use lignocellulose as feedstock. The crystal structure of two types of MCC (sugarcane pith and bamboo pith) and their derived carbon materials are studied, and the key findings are summarized as follows. (1) In the MCC refined from sugarcane pith, there are large amount of cellulose 2D crystal, which can be converted to valuable 2D graphene crystal. (2) In the MCC refined from bamboo pith there are large amount of cluster microcrystal cellulose, which can be converted to soft and elastic graphene microcrystal (GMC). (3) The 2D cellulose in MCC of sugarcane pith has large surface area and is easily to be degraded to sugars by acid–base hydrolysis reaction, which can be carbonized to Fullerenes-like carbon spheres. (4) The crystal structures of MCC derived carbon materials are strongly impacted by the crystal structures of MCC, and the carbonization reaction of MCC follows “in situ carbonization” and “nearby recombination” mechanism. In general, the results from this study may open a new way for value-added applications of microcrystal cellulose.

## Introduction

Carbon is one of the most active and important elements in the natural world, which has maintained a conserved cycle for billions years^1,2^. All living lives, including microorganism, plants, animals and human being, have existed in the carbon cycle for billion years^3,4^. However, the conserved carbon cycle has been badly disrupted by human economic activities during the past hundreds years with burning carbon-rich fossil fuels (coal, petroleum, and natural gas) in large scale, resulting in environmental pollution, ecology disorder, global warming, and frequently happened disaster climates^5,6^. On the other hand, biomass, including all the natural organic products from plants and microorganism on the earth and in water, is the most abundant and sustainable resource with a worldwide annual production about 8.5 × 10^10^ tons^7,8^, which contain huge amount of carbon, much more than the carbon consumed from fossil fuels each year. Biomass can play the central role in the battle to tackle greenhouse gas emissions and global warming^1,9–14^.

Carbon-based materials are among the most important materials that play many critical roles in current science and technology^8^. The importance of carbon-based materials has been recognized in recent decades by some of the highest scientific awards, including the 1996 Nobel Prize in Chemistry for fullerene^15^, the 2008 Kavli Prize in Nanoscience for carbon nanotubes^16,17^, and the 2010 Nobel Prize in Physics for graphene^18,19^. In recent decades carbon products has found important roles in anode materials of lithium ion battery and sodium ion battery, in electrode of supercapacitor^20–22^, and in carbon molecular sieves^23^ for gas separations of H_2_/CH_4_ and N_2_/O_2_^24–26^, and in molecular filter membrane for CO_2_ capture^25,27,28^, all are urgently needed for energy storage and transformation, and for environment and ecology protection. As a consequence, interest in carbon-based materials and their fabrication techniques is encountering most rapid development, and represents a very important topic in modern materials science.

Biomass has been the traditional resource for carbon materials for hundreds and thousands years in human history. However, the carbon products, directly produced from biomass, are often the low level products, such as wood charcoal and grass charcoal, without fine molecular structures. In order to make value-added carbon materials from biomass, varous new techniques has been developed in the past few decades^29–31^. For examples, after biomass is refined to cellulose, hemicellulose and lignin, 3D graphene microcrystal (GMC)^32^ and 2D graphene^33^ can be fabricated from lignin and celullose, respectively.

Microcrystal cellulose (MCC) is a part of lignocellulose in percentage around 10% to 30%^34–36^. The fine powder MCC is very different from the common fibrous lignocelluloses, and is often discarded in the industrial productions that use lignocellulose as raw material. In this article the structure of MCC and the carbon products derived from MCC are systematically studied. The results and conclusions from this study may provide a way for high-value utilization of the sustainable MCC resources.

## Materials and methods

In this study two types of MCC raw materials are used, one is taken from sugarcane pith^37,38^, and the other is taken from bamboo pith^39^. Both sugarcane pith and bamboo pith are wastes in pulp mills that use bagasse and bamboo as feedstock.

### Pretreatment of sugarcane pith

**Figure 1:**
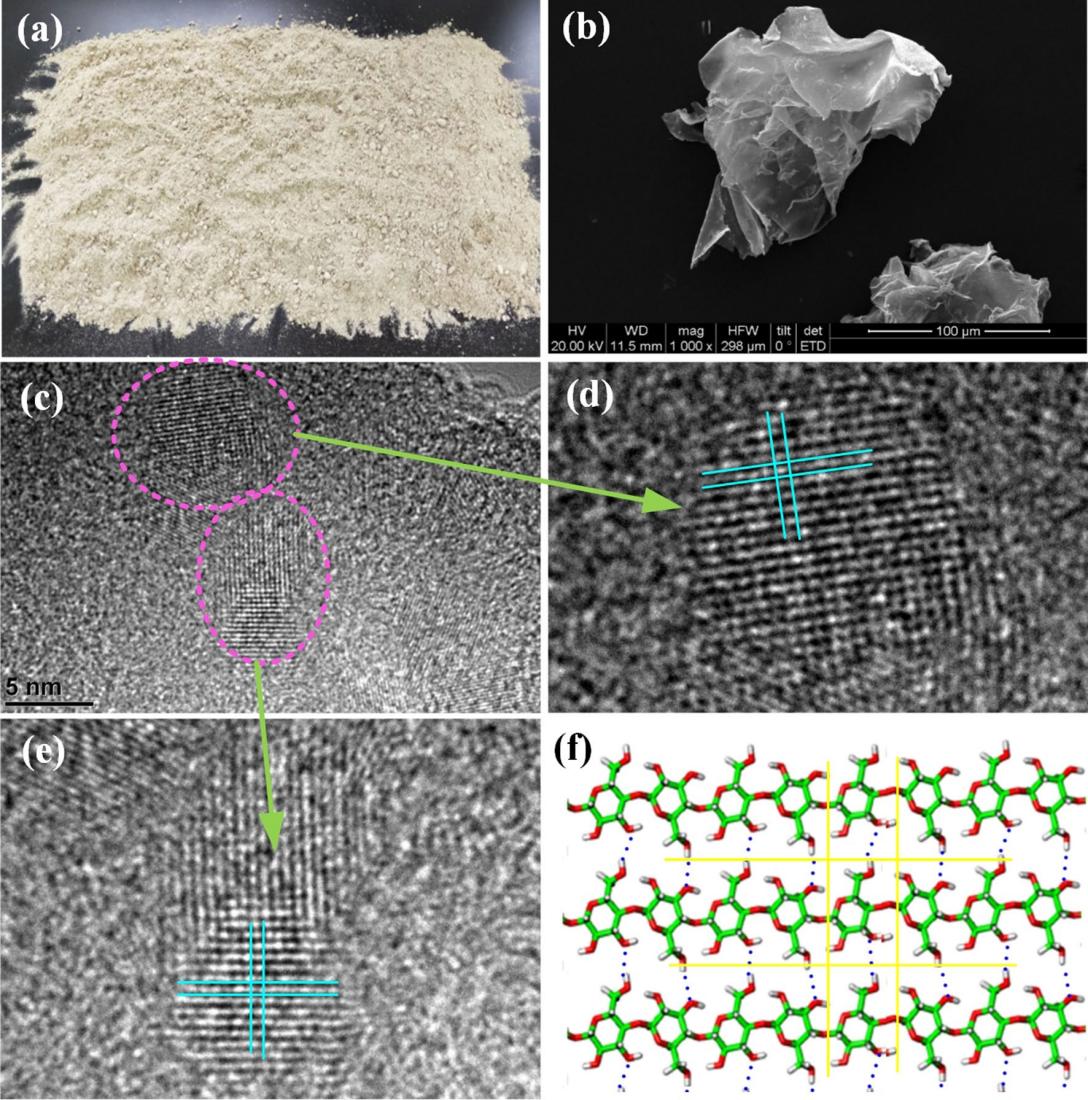
Sugarcane pith and its cellulose 2D crystal structure. (**a**) A photo of sugarcane pith separated from bagasse of a pulp mill in Nanning of Guangxi. (**b**) A SEM image of sugarcane pith after wind-selection. The powders of sugarcane pith are like dried petals of rose. (**c**) A HRTEM image of sMCC (sugarcane mcrocrystal cellulose refined from sugarcane pith). In the image cellulose 2D crystal structure can be seen clearly. (**d**) and (**e**) Close-view of two cellulose 2D crystals. (**f**) Molecular model of cellulose 2D crystal.

The fine powder of sugarcane pith is a part of sugarcane bagasse making up 30% of mass percentage^37,38^ that is very different from the bagasse fiber in appearance and structure. In the pulp mills using bagasse as feedstock, the sugarcane pith is firstly seperated and discarded, because it has no contribution to paper pulp, and consumes chemical reagents fruitlessly. Actually sugarcane pith is a complex collection, including various organs of sugarcane. The majority of sugarcane pith is the powder in form of dried rose petals that is a type of microcrystal cellulose (MCC). In this study the sugarcane pith discarded in pulp mill is further screened by using pneumatic separator, and in this way the impure components are removed and MCC is collected. Figure 1a is a photo of sugarcane pith provided by a bagasse pulp mill in Nanning of Guangxi, and Fig. 1b shows the SEM images of MCC of sugarcane pith after wind selection, where the powders of MCC are like dried petals of rose.

**Figure 2:**
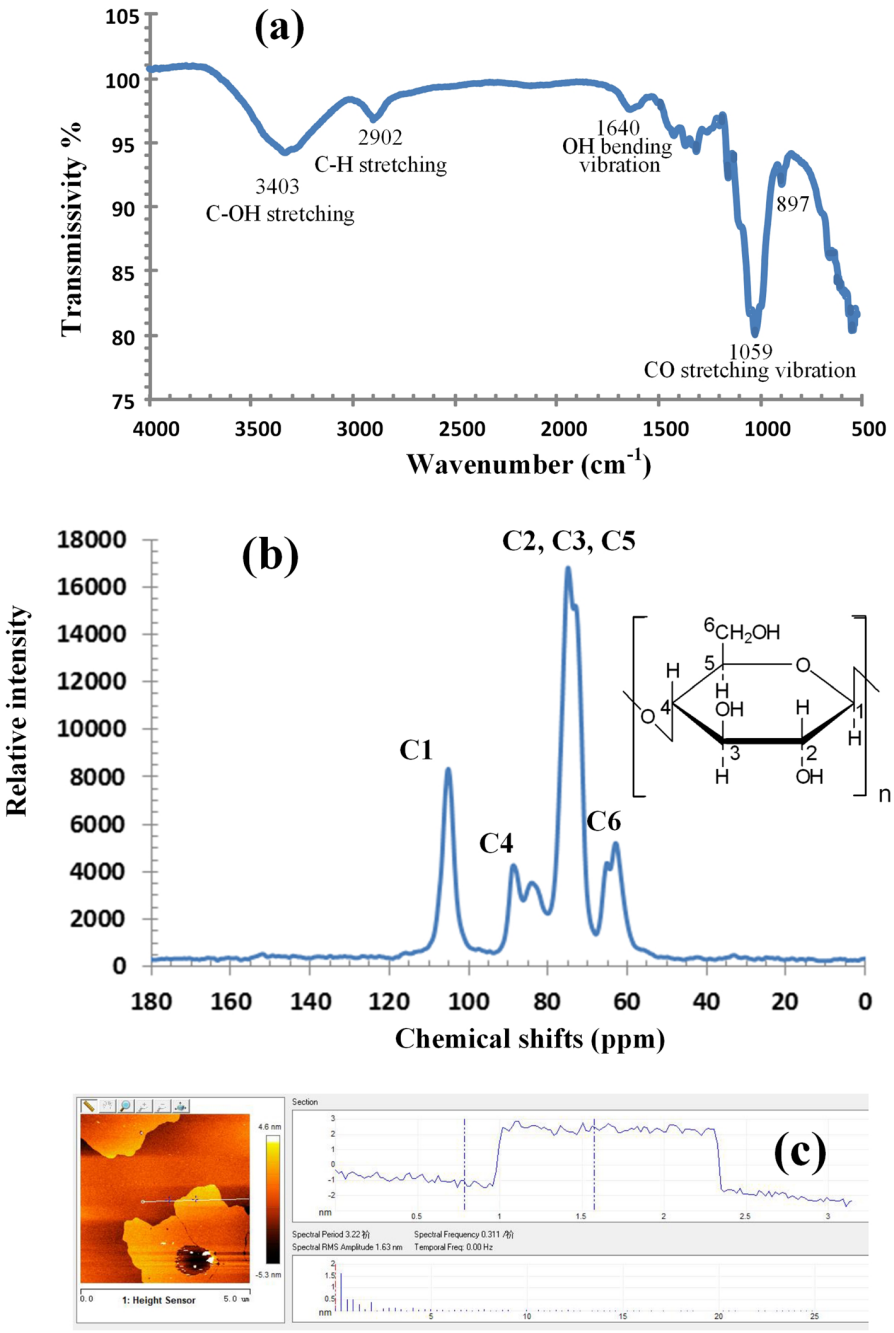
The FTIR spectrum, solid state ^1^H-NMR spectra and AFM spectrum of sMCC refined from sugarcane pith. (**a**) The FTIR spectrum of sMCC. (**b**) The solid state ^1^H-NMR spectra of sMCC. All the spectrums of FTIR and ^1^H-NMR indicate that sMCC refined from sugarcane pith is pure cellulose. (**c**) AFM spectrum of sMCC. The thickness of 2D cellulose is 2.5–3 nm.

In order to remove the lignin and hemicellulose, which chemically combined with MCC, the sugarcane pith is soaked in 10% NaOH solution and heated to 130 °C for 3 h, then washed by using deionized (DI) water, and dried in air drying oven. As refined sugarcane pith is labled as sMCC (sugarcane microcrystal cellulose), and Fig. 1c–e are the HRTEM images of sMCC, where the 2D crystal lattice of sMCC can be seen clearly, as indicated in Fig. 1d. Figure 1f is the molecular model of cellolose 2D crystal that is similar with the 2D crystal structures in Fig. 1d and e. The FTIR spectrum^40^ and solid state NMR spectra^35,41^ of sMCC are shown in Fig. 2a and b, respectively. Figure 2c is the AFM spectrum of sMCC, and the thickness of 2D cellulose is 2.5 to 3 nm. All the spectrums of FTIR, S-NMR, AFM and the images of SEM and HRTEM indicate that sMCC is pure cellulose with microcrystal structure in two dimensions.

### Pretreatment of bamboo pith

**Figure 3:**
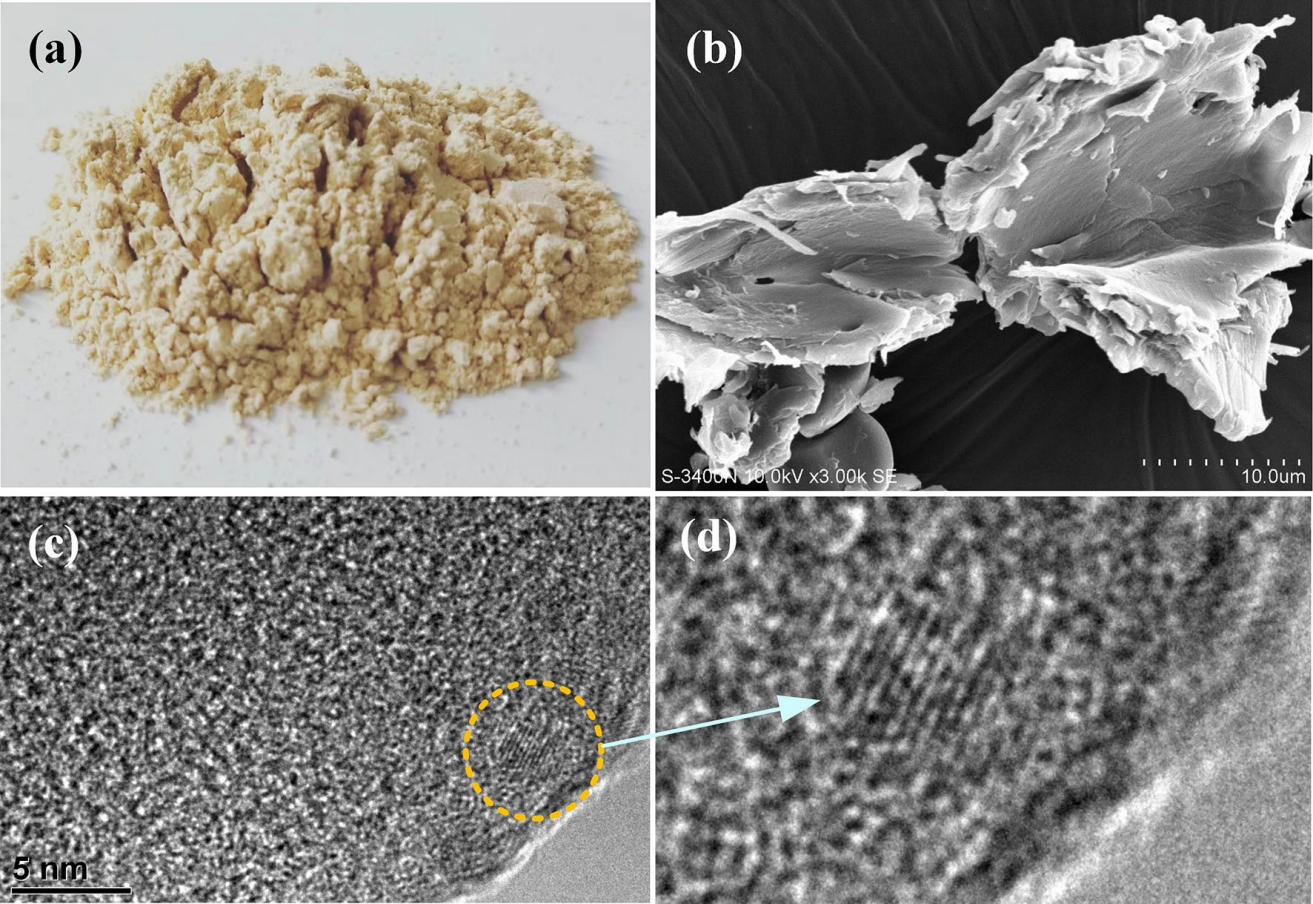
Bamboo pith and its cellulose microcrystal structure. (**a**) A photo of bamboo pith that is seperated and collected from crushed bamboo by using pneumatic separator. (**b**) A SEM image of bamboo pith. The powders of bamboo pith are like cabbage thick leaves. (**c**) A HRTEM image of bMCC (bamboo microcrystal cellulose). In the image a small cellulose crystal is spoted and marked in a circle. (**d**) A close view of the cellulose crystal. The lattice image shows the crystal lateral face of bMCC, having no two-dimensional feature, unlike the cellulose 2D crystal of sMCC.

In the pulp mills using bamboo as feedstock the bamboo is first crushed to pieces. During this process, a large amount of bamboo pith in fine powder form is produced around 20% in mass percentage, which comes from the iner layer of bamboo tube and the cross walls. If the fine powder of bamboo pith is not removed, it would eventually come into waste water, and cause high COD and pollution^42^. In this study the crushed pieces of bamboo are separated and segmented by using pneumatic separator, in this way the fine powder of bamboo pith is separated and collected. Figure 3a is a photo of bamboo pith, and Fig. 3b shows the SEM images of bamboo pith, where the powders of bamboo pith are like cabbage thick leaves, much thicker than the microcrystal cellulose (sMCC) of sugarcane pith in Fig. 1b. The lignin and hemiecellulose combined with microcrystal cellulose in bamboo pith is removed by using the same method as the sugarcane pith. As above purified bamboo pith is labled as bMCC (bamboo microcrystal cellulose). Figure 3c is a HRTEM image of bMCC, where a crystal structure of microcrystal cellulose is spoted and marked in a pink circle, and Fig. 3d is a close view of the crystal structure. The lattice image shows the crystal lateral face of bMCC, having no two-dimensional feature, unlike the cellulose 2D crystal of sMCC.

### Saccharification of MCC

**Figure 4:**
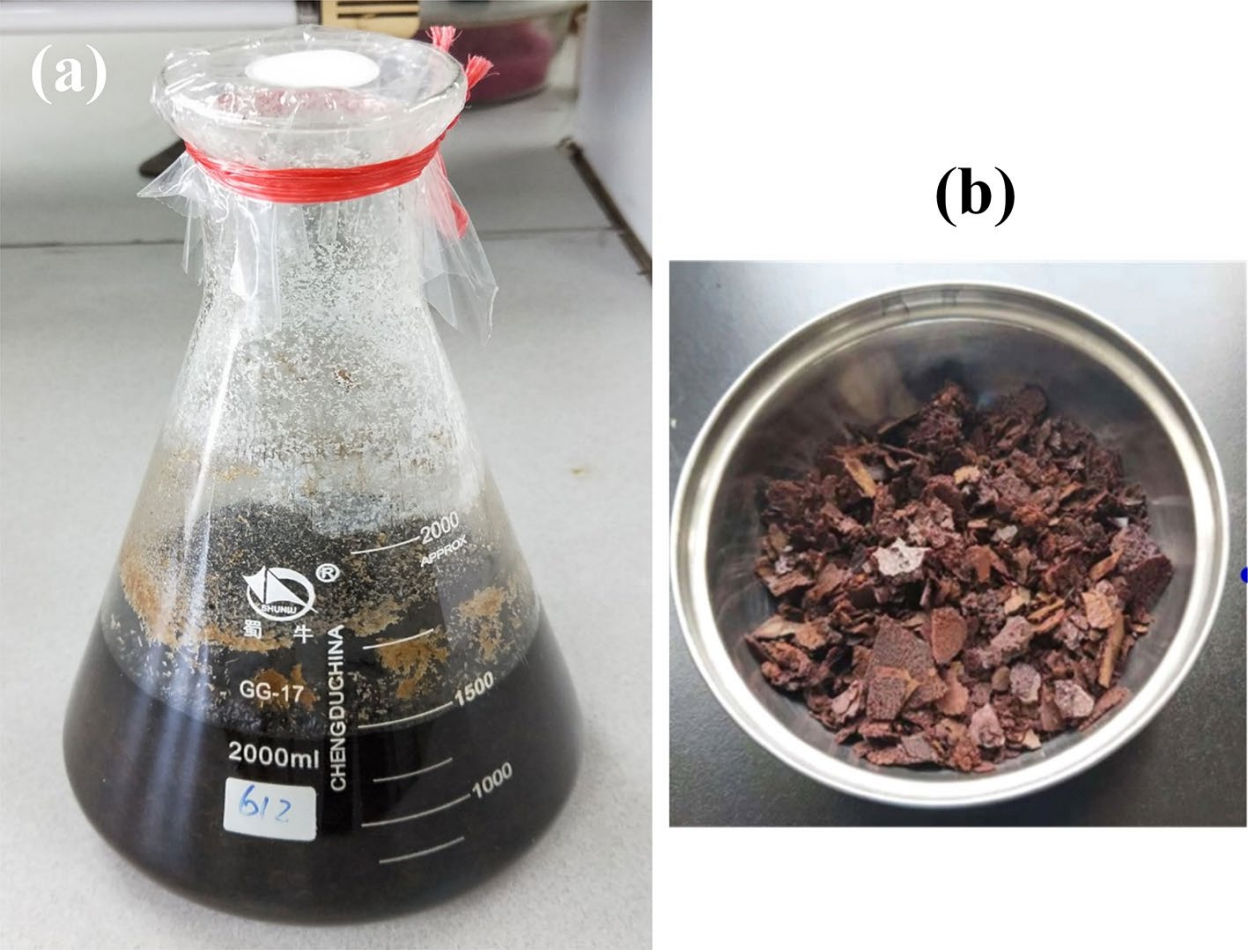
The photos of the saccharized sugarcane pith and sugar. (**a**) A photo of hydrolyzed sugarcane pith in phosphoric acid solution. (**b**) A photo of the sugar hydrolyzed from sugarcane pith. The remaining phosphoric acid in the dried sugar will play the role of actavation reagent in activation reaction.

Cellulose is a polymer of glucose monomers, which can be converted to sugars through acid–base hydrolysis reaction^37,43,44^. However, the hydrolysis reaction of common fibrous cellulose is very difficult because of its tight crystal structure. On the other hand the large and exposed surface area of microcrystal cellulose makes it easily to be saccharized and degraded to sugars with different degrees of polymerization. In this study the sMCC of sugarcane pith is soaked in phosphoric acid (H_3_PO_4_) solution of 5% mass concentration, and hydrolyzed at 90 °C for 6 h, then is dried in air-drying oven at 120 °C to solid. The sugar hydrolyzed as above is labeled as sMCCs. Figure 4a is a photo of the saccharized sugar sMCCs in solution, and Fig. 4b is a photo of the dried sugar sample (sMCCs). The phosphoric acid used for hydrolysis remains in dried sugar, which will play the role of activation reagent in the following carbonization and activation reaction of sMCCs.

### Carbonization of MCC precursors

The three MCC precursors (sMCC, bMCC and sMCCs), prepared as above, are carbonized in tube furnace in nitrogen atomosphere at temperature 800 °C to 1400 °C. For more exquisite graphene crystal structure higher temperature is needed. For fabrication of porous carbon product activation reagent is used in activation reaction. For facilitation of industrial production of MCC-derived carbon products, cheap and bulk biomass raw materials, common chemical reagents, and mature operation techniques are used in this study.

### Characterization methods

The MCC precursors and carbon products are measured and characterized using physical and chemical methods, including SEM (Scanning electron microscope), HRTEM (High Resolution Transmission Electron Microscope), FTIR (Fourier transform infrared spectroscopy), S-NMR (Solid-state nuclear magnetic resonance), AFM (Atomic force microscope), BET (Brunauer, Emmett and Teller) Gas adsorption instrument, and XPS (X-ray photoelectron spectroscopy).

The SEM images are taken by using an instrument Hitachi S-3400. In order to obtain clearer SEM images, the samples are first coated by gold-beam. The crystal structures of MCC samples and derived carbon products are characterized by the HRTEM. In this study the HRTEM images are taken by a commercial cororation (Tianhe (Shandong) Testing Technology Co, Ltd, https://www.keysci.com/) using Transmission Electron Microscope (Tecnai G2 F30). The Minimum Dose Sytem and other helpful tools are integrated into this instrument. In order to avoid samples to be damaged, when the HRTEM images of MCC samples are taken, the irradiation intensity and imaging conditions are caerfuly adjusted and selected. The chemical structures of MCC samples are identified by using FTIR instrument (Thermo fisher Nicolet IS10) and solid-state NMR (Direct Drive 2, Agilent). The surface of 2D crystal cellulose samples are measured and characterized by using AFM (NX10, Park Systems). The specific surface area and pore structure of active carbon is measured and characterized by using Automatic Volumetric Sorption Analyzer (Autosorb-1MP, Quantachrome) and BET method. The atomic composition and electron states on the surface of MCC precursous and derived carbon products are analyzed by using XPS (PHI Quantera II).

## Results and discussion

In this section the three MCC precursors are first carbonized in tube furnace at temperature 800 °C to 1400 °C in nitrogen atomosphere, respectively, then measured and characterized.

### Carbonization of sMCC

**Figure 5:**
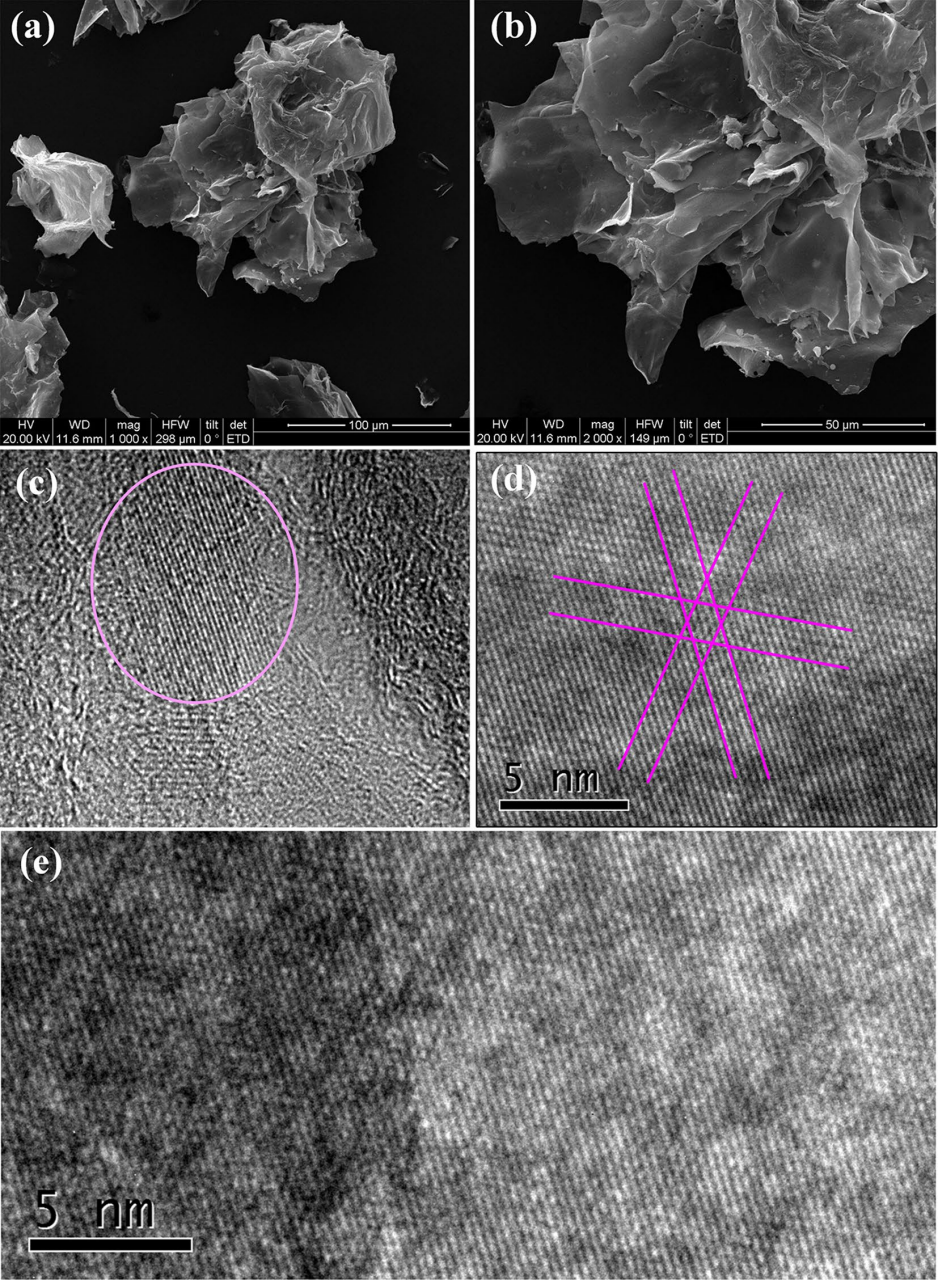
The SEM and HRTEM images of 2D graphene derived from cellulose 2D crystal of sMCC (sugarcane microcrystal cellulose). (**a**) The SEM image of 2D graphene derived from 2D cellulose. (**b**) A close-view of a 2D graphene particle. The particles of 2D graphene are like dried petals of rose, very similar to its precursor. (**c**) The HRTEM image of 2D graphene. (**d**) A close view of the graphene 2D crystal. (**e**) A larger graphene 2D crystal. Exquisite honeycomb-shaped hexagonal lattice can be seen clearly. Careful calculation reveals that the sides length of the hexagon is 0.143 nm. This is exactly the crystal structure of 2D graphene.

The carbon product, carbonized from sMCC at tempereture1400 °C for 1 h, is labeled as sMCC-C. A notable phenomenon is that the sMCC-derived carbon sMCC-C keeps the shape and architecture of its precursor sMCC. The SEM images of sMCC-C in Fig. 5a and b are like dried petals of rose, very similar to its precursor sMCC in Fig. 1b.

A 10 nm resolution HRTEM image of sMCC-C is shown in Fig. 5c, in which a large patch of graphene 2D crystal is marked in pink circle, and Fig. 5d and e are the close view of the graphene 2D crystal. In Fig. 5d there are three groups of parallel lines foming 120° angle. After careful calculation the side length of the hexagon units is 0.143 nm, and this is exactly the side length of graphene. According to the rule of “in situ carbonization”, the graphene 2D crystal structure of sMCC-C in turn proves the two-dimensional crystal structure of precursor sMCC. The Raman spectrum of sMCC-C is shown Fig. 8, where the peak-D at 1339 cm^−1^, peak-G at 1564 cm^−1^, peak-2D at 2677 cm^−1^, and peak-(D + G) at 2901 cm^−1^ indicate that there is a certain amount of graphene in sMCC-C, although not all particles in sMCC-C are 2D graphene.

The 2D graphene crystal converted from 2D cellulose crystal was firstly reported by Du’s research team^33^, where the cellulose 2D crystal was produced through deep hydrolysis of bagasse fibrous cellulose in NaOH solution. In this study the cellulose 2D crystal is found from sugarcane pith, and is converted to graphene 2D crystal. A reasonable inference is that 2D graphene can be fabricated from 2D cellulose precursor, and this may open a new way for production of 2D graphene from sustainable microcrystal cellulose in very low cost.

### Carbonization of bMCC

**Figure 6:**
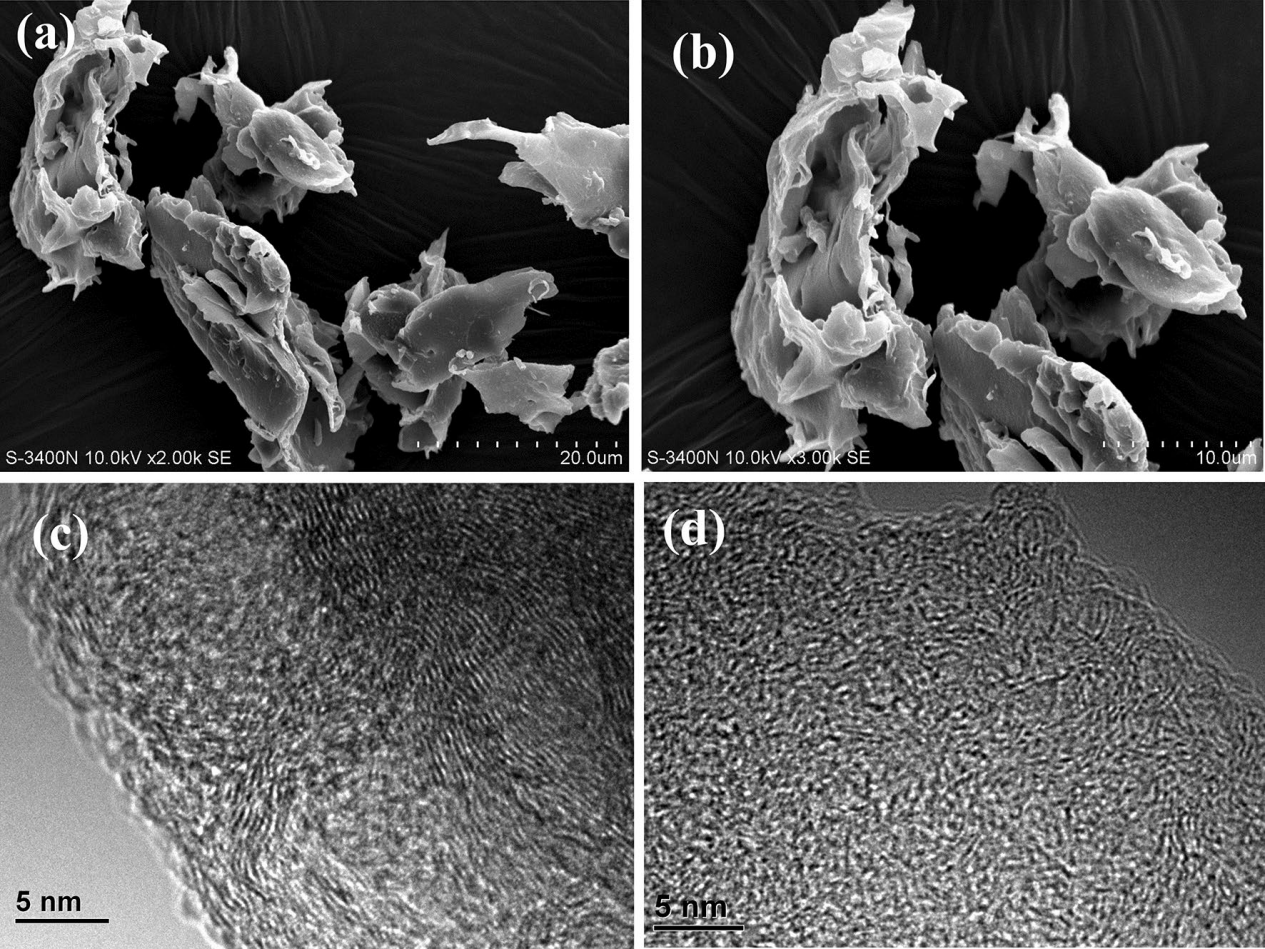
The SEM and HRTEM images of graphene microcrystal (b-GMC) derived from bMCC (bamboo microcrystal cellulose). (**a**) The SEM image of b-GMC derived from bMCC. (**b**) A SEM close-view of b-GMC derived from bMCC. The particles of GMC are like thick cabbage leaves, similar with their precursor. (**c**) A HRTEM image of bMCC derived b-GMC. The image is filled by multiple layers graphene microcrystals in different directions. (**d**) A different HRTEM image of bMCC derived b-GMC. In this image the b-GMC particles are even smaller, only a few nm and a few layers. The bMCC derived b-GMC is soft and elastic, very different from the lignin derived hard l-GMC.

The carbon product, carbonized from bMCC at tempereture1400 °C for 1 h, is labeled as bMCC-C that is fluff-like mass of soft and elastic. Figure 6a is a SEM image of bMCC-C powder, and Fig. 6b is a close-view of a bMCC-C particle, where the round shape is like its precursor bMCC in Fig. 3b.

Figure 6c is a HRTEM image of bMCC-C, where the multiple parallel short lines are the lateral faces of graphene microcrystals (GMC) that distribute on different directions. In Fig. 6c there is no visible graphene 2D crystal, very different from sMCC-C in Fig. 5. Another HRTEM image of bMCC-C is shown in Fig. 6d, where the particles of graphene microcrystal (GMC) are even smaller than that in Fig. 6c, only a few nm and a few layers. In a research article of Du’s team^45^, GMC (graphene microcrystal) fabricated from lignin was reported that is a type of hard carbon like glass and ceramic^29,46–48^, however, the GMC derived from bMCC is soft and elastic. A comparison between bMCC-derived GMC (labeled as b-GMC) and lignin-derived GMC (labeled as l-GMC) is shown in Fig. 10, we will discuss it more detailed in theoretical analysis section.

### Carbonization and activation of sMCCs

**Figure 7:**
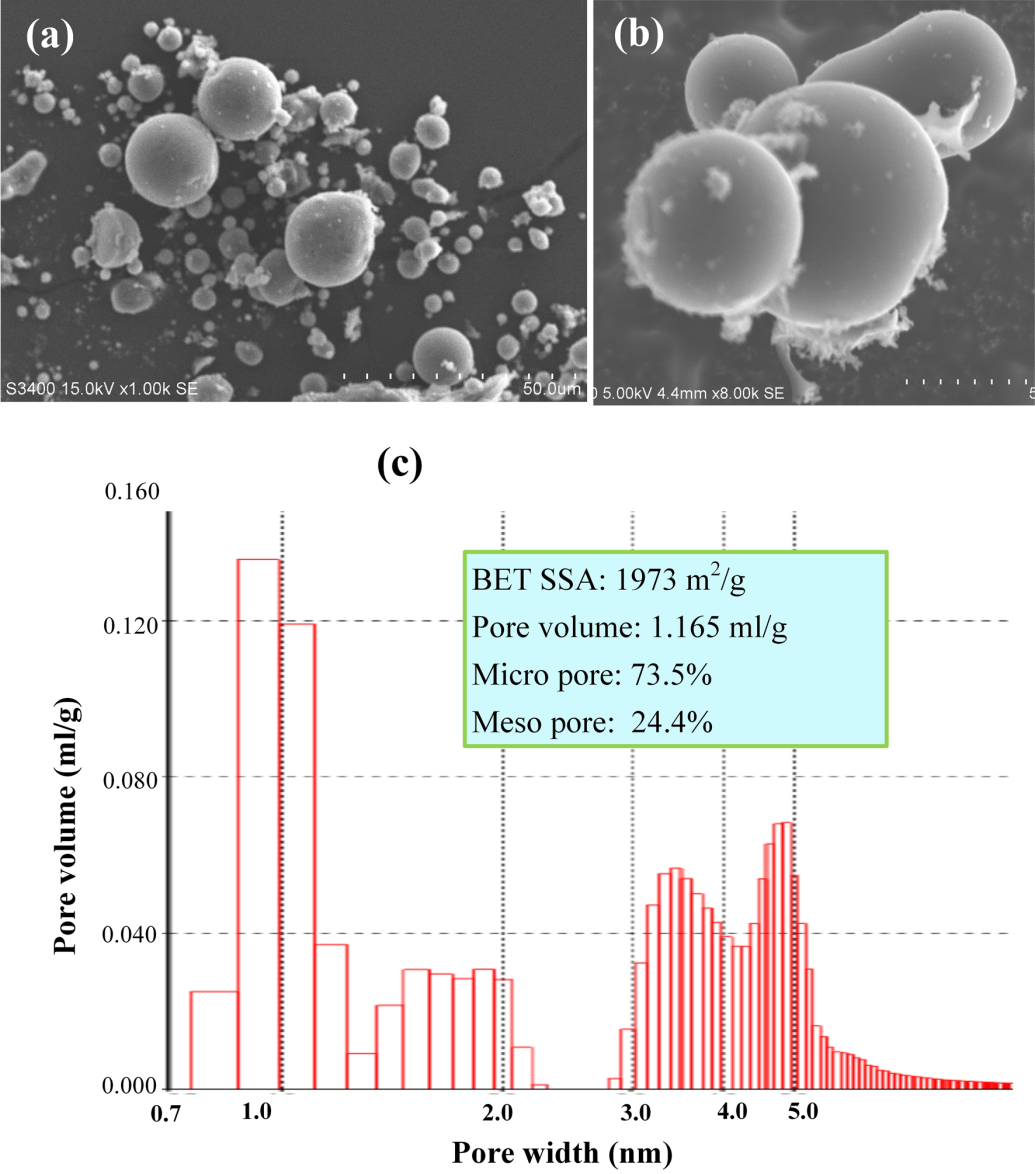
SEM images and pore size distribution of sphere porous carbon derived from sugar hydrolyzed from sugarcane pith. (**a**) The SEM image of sphere carbon. (**b**) A SEM close-view of sphere carbon. The diameters of sphere carbon particles are from 5 to 20 micron. (**c**) The distribution curve of pore-volume vs pore-size. In the sphere porous carbon micro pore volume is 73.5% and the mesoporous pore volume is 24.4%. The specific surface area (SSA) of sphere porous carbon is 1973 m^2^/g, and the total pore volume is 1.165 ml/g, a good porous carbon for supercapacitor.

Spherical carbon and fullerene carbon dot^49,50^ is usually fabricated from sugar, starch, and relative sugar polymers^51–53^. In this study the sMCCs sugar, hydrolyzed from sugarcane pith, is used to fabricate the sphere carbon. The saccharified sMCCs precursor with residual phosphoric acid is put in graphite crucibles, carbonized in a tube furnace in nitrogen atmosphere at tempereture 800 °C for 1 h, where phosphoric acid plays the role of activation reagent. The produced carbon is washed using DI water to neutral, then dried. As fabricated sphere carbon is labeled as sMCCs-C. Figure 7a and b are the SEM images of sMCCs-C, where the sphere carbon particles are in diameter 5 to 20 microns.

The porous sphere carbon sMCCs-C was activated by phosphoric acid in the carbonization reaction. The specific surface area (SSA) of sMCCs-C is 1973 m^2^/g, and the total pore volume is 1.1653 ml/g, measured by using BET (Brunauer–Emmett–Teller) gas adsorption method. The curve of pore-volume *vs* pore-size is shown in Fig. 7c, in which the micro pore volume is 73.5% and the mesoporous pore volume is 24.4%, a good porous carbon for supercapacitor^20–22^.

### Characterization and theoretical analysis

**Figure 8:**
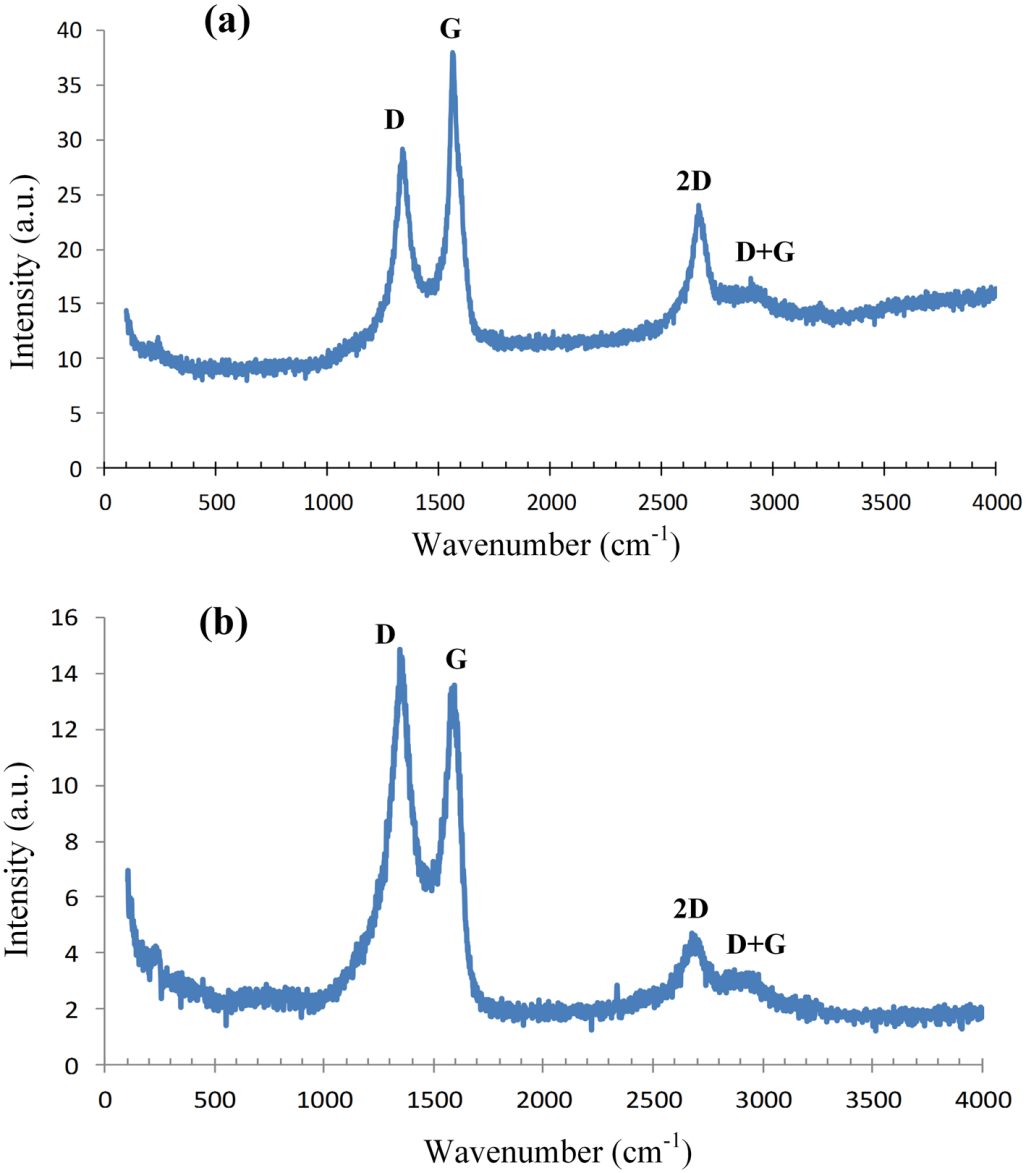
The Raman spectrums of sugarcane pith derived carbon and bamboo pith derived carbon. (**a**) The Raman spectrum of sMCC (sugarcane mcrocrystal cellulose) derived carbon. (**b**) The Raman spectrum of bMCC (bamboo mcrocrystal cellulose) derived carbon. The peak-D at 1339 cm^−1^, peak-G at 1564 cm^−1^, peak-2D at 2677 cm^−1^, and peak-(D + G) at 2901 cm^−1^ indicate that there is a certain amount of graphene in the mcrocrystal cellulose (MCC) derived carbon, althou not all carbon particles are graphene.

In this study the 2D graphene is derived from 2D cellulose of sMCC and the graphene microcrystal (GMC) is derived from bMCC. The HRTEM images in Fig. 5 are the direct evidence of sMCC-derived 2D graphene, and the HRTEM images in Fig. 6 are the direct evidence of bMCC-derived GMC. However, not all ingredients in sMCC-C and bMCC-C are graphene. Raman spectroscopy is a powerful tool for characterization of graphene and relative materials. The Raman spectrums of sMCC-C and bMCC-C are shown in Fig. 8a and b, respectively, where the peak-D at 1339 cm^−1^, peak-G at 1564 cm^−1^, peak-2D at 2677 cm^−1^, and peak-(D + G) at 2901 cm^−1^ indicate that there is a certain amount of graphene in the MCC-derived carbon products, althou not all carbon particles are graphene.

An explanation for the chemical reaction from cellulose 2D crystal to graphene 2D crystal is as follows. The carbonization reaction of cellulose 2D crystals follows the mechanism of "in situ carbonization" and "nearby recombination". According to this mechanism, the 6-carbon glucose monomers of 2D cellulose lost water molecules at high temperature, recombine into benzene rings, and form the 2D crystal structure of graphene on the basis of the precursor’s 2D crystal structure,

$$\left( {\text{2D cellulose}} \right)\left( {{\text{C}}_{{6}} {\text{H}}_{{{1}0}} {\text{O}}_{{5}} } \right)_{{\text{n}}} \to \left( {\text{2D graphene}} \right)\left( {{\text{C}}_{{6}} } \right)_{{\text{n}}} + {\text{ 5n}}\left( {{\text{H}}_{{2}} {\text{O}}} \right).$$

The 2D crystal cellulose has a large exposed surface area and many hydrogen bond donors and acceptors that are not bonded, therefore it is unstable and usually cannot exist independently. However, in the high concentration sucrose solution in the sugarcane tube, the hydrogen bond elements on the glucose monomer of 2D cellulose were bonded with sucrose and played a stabilizing role. Consequently 2D crystal cellulose could exist in sugarcane pith.

**Figure 9:**
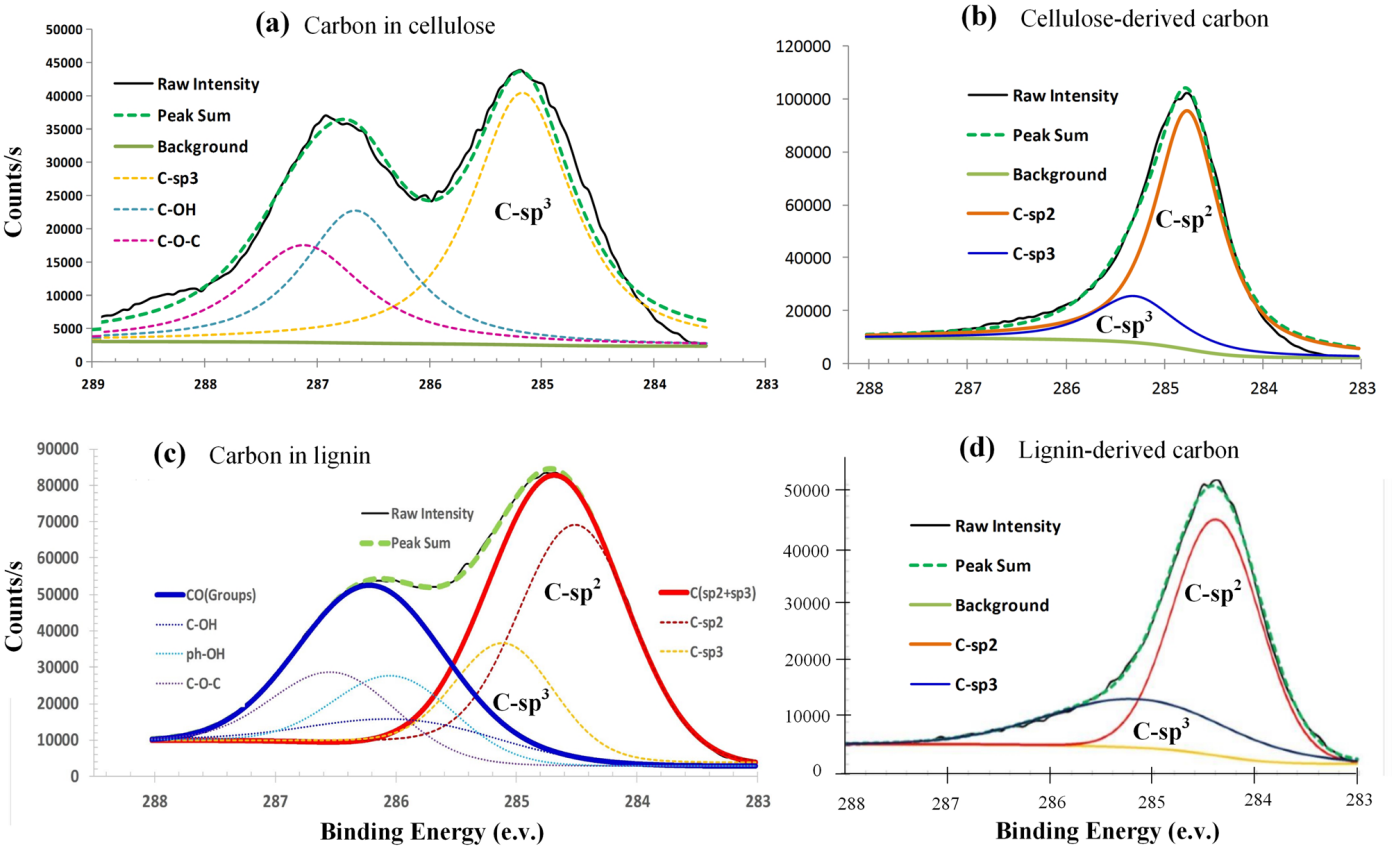
The XPS spectrums of bMCC (bamboo mcrocrystal cellulose), lignin, bMCC-derived carbon (bMCC-C), and lignin-derived carbon (lignin-C). (**a**) The XPS spectrum of carbon atoms in bMCC. The sub-peak centered on 285.2 nm is of the sp^3^ carbon atoms. (**b**) The XPS spectrum of carbon atoms in bMCC-C is in sp^2^ state (284.7 nm). Most carbon atoms change their electron state from sp^3^ in bMCC to sp^2^ in bMCC-C (graphene microcrystal, b-GMC) during carbonization reaction. (**c**) The XPS spectrum of carbon atoms in lignin. The sub-peak centered on 284.7 nm is of carbon atoms, where most carbon atoms are in sp^2^ state. (**d**) The XPS spectrum of carbon atoms in lignin-derived graphene microcrystal (l-GMC). Most carbon atoms keep their original electron states (sp^3^ or sp^2^) in lignin during carbonization reaction.

Following the same reaction mechanism the cabbage leaves like cellulose microcrystals of bamboo pith are converted to graphene microcrystal (b-GMC) keeping the shape of its precursor bMCC. The bMCC derived b-GMC is soft and elastic, very different from the lignin-derived l-GMC^45^, the latter is very hard, like glass and ceramic. The XPS spectrum of carbon atoms in bMCC is shown in Fig. 9a, where the sub-peak-1 centered on 285.2 nm is of carbon sp^3^ atoms, and the sub-peak-2 centered on 286.8 nm is of carbon atoms bonded by oxygen atoms. Figure 9b is the XPS spectrum of carbon atoms of bMCC-C. An interesting phenomenon is that in the sub-peak-1 of carbon of Fig. 9a almost all carbon atoms of bMCC are in sp^3^ electron state (285.2 nm), however, in Fig. 9b most carbon atoms of bMCC-C are in sp^2^ electron state (284.7 nm). It means that during carbonization reaction most carbon atoms of bMCC are converted their electron state from sp^3^ in cellulose to sp^2^ in graphene, and the other carbon atoms are in an amorphous form, keeping their sp^3^ electron state.

**Figure 10:**
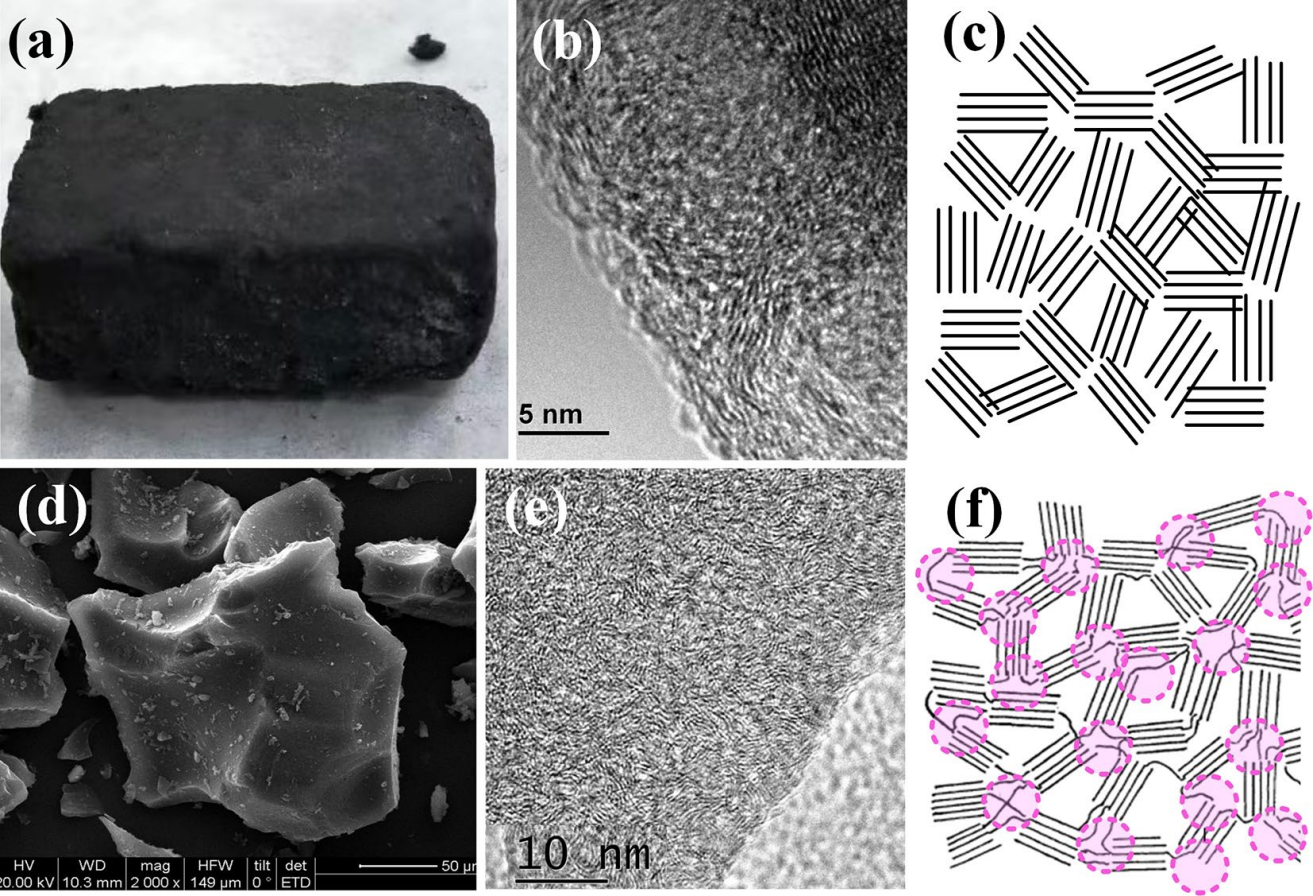
Comparison of bMCC-derived b-GMC and lignin-derived l-GMC. (**a**) A photo of bMCC-derived b-GMC. (**b**) A SEM image of bMCC-derived b-GMC. The b-GMC is soft and elastic. (**c**) Molecular model of bMCC-derived b-GMC. (**d**) A SEM image of lignin-derived l-GMC. The l-GMC is hard, like glass and ceramic. (**e**) A HRTEM image of l-GMC. (**f**) Molecular model of lignin-derived GMC. In lignin-derived l-GMC the sp^2^ graphene microcrystal units are chemically bonded by sp^3^ carbon atoms, forming the sp^2^-sp^3^ hybrid hard GMC. On the other hand, the micrystal units of b-GMC are randomly piled together forming a soft and elastic aggregate without chemical bond connection.

The XPS spectrums of lignin and lignin-derived carbon are shown in Fig. 9c and d, respectively. Based on the XPS spectrum of lignin in Fig. 9c, 72.7% carbon atoms are in sp^2^ state and 27.3% carbon atoms are in sp^3^ state. And based on the XPS spectrum of lignin-derived carbon (l-GMC) in Fig. 9d, the percentages of sp^2^ and sp^3^ carbon atoms are around 73% and 27%, respectively, almost the same as the bMCC-derived b-GMC. In the lignin-derived l-GMC the sp^2^ graphene microcrystals are chemically bonded by sp^3^ carbon atoms, forming the sp^2^-sp^3^ hybrid hard graphene microcrystal, like glass and ceramic. On the other hand, in bMCC-derived b-GMC the sp^3^ carbon atoms are in amorphous form, and the sp^2^ graphene microcrystals are piled together without chemical bond conection. Figure 10 is a comparison of bMCC-derived b-GMC and lignin-derived l-GMC. Both b-GMC and l-GMC consist of microcrystal graphene units. However, in b-GMC the graphene micrystal units are randomly piled together forming a soft and elastic aggregate, while in l-GMC the graphene micrystal units are chemically bonded by sp^3^ carbon atoms forming very hard carbon block. The model structures of b-GMC and l-GMC in Fig. 10c and f well illustrate the structural difference between b-GMC and l-GMC. In Fig. 10f the pink circles are the sp^3^ carbon atoms that join the graphene microcrystal units by chemical bonds, while in Fig. 10c the micrystal units of b-GMC are randomly piled together forming a soft and elastic aggregate without chemical bond connection.

## Conclusion

In this study the carbon materials, derived from two types of microcrystal cellulose (MCC), sugarcane pith and bamboo pith, are systmetically studied, including pretreatment of MCC, fabrication and characterization techniques of carbon materials, and theoretical analysis of reaction mechanism. Some of the key findings from this study are summarized as follows. Microcrystal cellulose is a green and sustainable resource, makes up 30% and 20% mass percentage in sugarcane bagasse and bamboo, respectively, which can be separated and collected efficiently by using pneumatic separator. After lignin and hemicellulose are removed from sugarcabe pith and bamboo pith by means of biorefinary technique, very pure MCC can be obtained that are good precursors for value-added carbon materials. In the sugarcane pith refined sMCC, there are large amount of cellulose 2D crystal, which can be converted to high-value 2D graphene crystal by carbonization reaction at high temperature. In the bamboo pith refined microcrystal cellulose bMCC there are large amount of thick microcrystal cellulose, which can be converted to soft and elastic graphene microcrystal (b-GMC) by carbonization reaction at high temperature, very different from the lignin-derived hard graphene microcrystal (l-GMC). The MCC powder has large surface area and is easily to be degraded to sugars with different degrees of polymerization of glucose monomers by acid–base hydrolysis reaction, which can be carbonized to Fullerenes-like carbon spheres at high temperature. Carbonization reaction of MCC follows “in situ carbonization” and “nearby recombination” mechanism, consequently the crystal structures and architectures of MCC-derived carbon products are strongly impacted by that of their MCC precusors. Overall, the results and conclusions of this study may initiate a new way for value-added applications of microcrystal cellulose, which once was discarded or lost with waste water and caused high COD and pollution in the plants using lignocellulosic feedstock.

## Data Availability

All data that support the findings in this study are in the article, and asking for detailed experimental data is possible depending on further communication.
